# Cellular mechanisms of fibrin (ogen): insight from neurodegenerative diseases

**DOI:** 10.3389/fnins.2023.1197094

**Published:** 2023-07-17

**Authors:** Tingting Wen, Zhaohui Zhang

**Affiliations:** Department of Neurology, Renmin Hospital, Wuhan University, Wuhan, China

**Keywords:** fibrinogen, fibrin, CNS, neurodegenerative disease, AD, MS

## Abstract

Neurodegenerative diseases are prevalent and currently incurable conditions that progressively impair cognitive, behavioral, and psychiatric functions of the central or peripheral nervous system. Fibrinogen, a macromolecular glycoprotein, plays a crucial role in the inflammatory response and tissue repair in the human body and interacts with various nervous system cells due to its unique molecular structure. Accumulating evidence suggests that fibrinogen deposits in the brains of patients with neurodegenerative diseases. By regulating pathophysiological mechanisms and signaling pathways, fibrinogen can exacerbate the neuro-pathological features of neurodegenerative diseases, while depletion of fibrinogen contributes to the amelioration of cognitive function impairment in patients. This review comprehensively summarizes the molecular mechanisms and biological functions of fibrinogen in central nervous system cells and neurodegenerative diseases, including Alzheimer’s disease, Multiple Sclerosis, Parkinson’s disease, Vascular dementia, Huntington’s disease, and Amyotrophic Lateral Sclerosis. Additionally, we discuss the potential of fibrinogen-related treatments in the management of neurodegenerative disorders.

## 1. Introduction

Fibrinogen is a complex, fibrous glycoprotein synthesized mostly by hepatocytes in the liver (Kattula et al., 2017). Normally, fibrinogen concentration in blood is about 2–4 g/L, with a half-life of approximately 4 days (Tennent et al., 2007). Fibrinogen is primarily found in plasma and is typically far from the brain parenchyma (Galea, 2021). However, in conditions such as inflammation-associated diseases, the concentration of fibrinogen increases rapidly, and it is converted to fibrin by the action of thrombin, which plays a role in blood coagulation (Davalos and Akassoglou, 2012). Thus, Fibrinogen is considered an acute-phase protein. The synthesis of fibrinogen is regulated by hormones and cytokines. The glucocorticoids dexamethasone and interleukin-6 (IL-6) increase fibrinogen biosynthesis, while estrogen (Estadil-17) and inflammatory cytokines (IL-4, IL10, IL13) decrease fibrinogen biosynthesis (Weisel, 2005). Fibrinogen contains different binding sites in different parts of the molecule that play essential roles in coagulation, inflammation, and tissue repair. By binding to different receptors on the surface of cells in the hematopoietic, immune, and nervous systems, fibrinogen plays a critical role in regulating a range of biological functions. In recent years, research has focused on the role of fibrinogen in the aging process, as its levels have been found to increase with age and are associated with several age-related diseases, such as stroke, cardiovascular disease, and dementia. Understanding the mechanisms by which fibrinogen contributes to these diseases may lead to the development of novel therapies to prevent or treat age-related illnesses.

Neurodegenerative diseases encompass a spectrum of disorders characterized by diverse clinical symptoms and underlying molecular mechanisms. These conditions commonly exhibit cognitive deficits, memory impairment, motor dysfunction, and the accumulation and propagation of pathological proteins (Ga et al., 2018). Additionally, inflammation and oxidative stress play crucial roles in their progression. Recent investigations have unveiled the presence of fibrinogen and other blood factors in the brains of individuals afflicted with neurodegenerative diseases, implicating their involvement in triggering innate immune responses and neuroinflammation (Jeon et al., 2021). Moreover, fibrinogen possesses the capability to interact with receptors in cells of the central nervous system (CNS), thereby activating signaling pathways that influence fundamental cellular functions and impact inflammatory, neurodegenerative, and reparative processes associated with these diseases (Adams et al., 2004, 2007b; Davalos and Akassoglou, 2012; Bardehle et al., 2015). Scientific studies have demonstrated that fibrinogen exacerbates oxidative stress and inflammatory responses in neurodegenerative disorders, while depletion of fibrinogen can ameliorate cognitive impairment in affected individuals. Within this review, we elucidate the structural aspects of fibrinogen and its molecular mechanisms within the CNS. Additionally, we explore the effects and underlying mechanisms through which fibrinogen impacts neurodegenerative diseases. Furthermore, we provide a comprehensive summary of the current progress in utilizing fibrinogen-related drugs for the treatment of these devastating conditions.

## 2. Structure of fibrinogen

Fibrinogen, a soluble glycoprotein, is a vital component of the coagulation cascade. It has a large molecular weight of 340 kDa and is comprised of three polypeptide chains: Aα, Bβ, and γ. These chains are encoded by gene clusters on human chromosome 4. Fibrinogen’s primary function is to convert into fibrin during clot formation, preventing excessive bleeding (Kattula et al., 2017). The individual molecular weights of the Aα, Bβ, and γ chains are 66.5 KDa, 52 KDa, and 46.5 KDa, respectively (Zhmurov et al., 2016). All six chains of fibrinogen are held together by 29 disulfide bonds, resulting in two identical half-molecules. Its structural morphology exhibits a rod-like shape measuring 45 nm in length and 2–5 nm in thickness (Hall and Slayter, 1959; Weisel et al., 1985). Its Stokes-Einstein radius is approximately 8.4 nm (Potschka, 1987). While the majority of fibrinogen’s structure has been elucidated through X-ray crystallography, certain components, such as the αC region, remain unresolved. The αC region, located at the C-terminus of the α-chain, extends toward the center of fibrinogen and interacts with its central region (Litvinov et al., 2007). Studies have shown that modifications to the αC region can affect fibrin formation, leading to the development of longer, thinner fibrils, and denser fibrin webs (Medved and Weisel, 2022). These findings highlight the critical role of the αC region in regulating fibrin’s structural and mechanical properties, with potential implications for age-related changes in blood clotting and associated diseases. Fibrinogen consists of two globular D regions and one globular E region, named after their corresponding proteolytic fragments. The D region, situated at the N-terminus of the α, β, and γ chains, contains seven domains that contribute to the α-helix coil structure. Within the D region, the β nodule and γ nodule are formed by the N-termini of the β and γ chains, respectively, with the γ nodule playing a pivotal role in fibrin formation. On the other hand, the E region comprises four domains derived from the C-terminus of the α, β, and γ chains. It forms a coiled-coil structure consisting of a triple α-helical arrangement. The central nodule, a globular portion within the E region, lacks coiled coils (Medved and Weisel, 2009). These distinct domains of fibrinogen interact with specific cellular receptors, thereby exerting diverse functions.

## 3. Function and mechanism of fibrinogen in the CNS

Fibrinogen, a complex protein, possesses diverse functions beyond blood clotting. It plays roles in inflammation, tissue repair, and cancer development (Davalos and Akassoglou, 2012). In the nervous system, fibrin(ogen) has been found to have detrimental effects, such as activating microglia, damaging axons, inhibiting the differentiation of Schwann cells and oligodendrocyte progenitor cells, impairing myelin repair, inducing astrocytic scar formation, and hindering neurite outgrowth (Petersen et al., 2018). Fibrin(ogen) interacts with specific receptors expressed by various nervous system cells and proteins that regulate important nervous system functions. Its effects can occur through receptor binding or direct interaction with substrates.

The interaction between fibrinogen and cellular prion protein (PrP^C^) has been found to induce the overexpression of tyrosine receptor kinase B (TrkB) in astrocytes, triggering the production of reactive oxygen species (ROS) and nitric oxide (NO). These oxidative stress-inducing molecules can contribute to neurodegeneration (Colombo et al., 2012; Charkviani et al., 2020). Additionally, fibrinogen binding to astrocyte receptors like intercellular adhesion molecule-1 (ICAM-1) or PrP^C^ has been shown to increase the expression of inflammatory cytokines such as IL-6, C-X-C motif chemokine ligand 10 (CXCL-10), and C-C motif chemokine ligand 2 (CCL2), as well as the production of ROS and NO in astrocytes, leading to oxidative stress and astrocyte death (Sulimai et al., 2021b). Fibrinogen may exacerbate neuroinflammation by upregulating IL-6 expression in astrocytes, creating a positive feedback loop where IL-6 stimulates fibrinogen synthesis, further amplifying IL-6 expression and perpetuating chronic neuroinflammation (Vasse et al., 1996). Furthermore, fibrinogen, whether in soluble or matrix-associated form, can carry an inactive form of transforming growth factor-β (TGFβ), which becomes activated upon encountering primitive astrocytes. This activation leads to Smad2 phosphorylation in astrocytes, inhibiting neurite outgrowth (Schachtrup et al., 2010).

Research indicates that fibrinogen can hinder neurite outgrowth by acting as a ligand for the β3 integrin receptor on neurons. This interaction triggers the aggregation and phosphorylation of the EGF receptor (EGFR), further inhibiting neurite outgrowth. However, inhibiting the binding between fibrinogen and β3 integrin or suppressing EGFR phosphorylation effectively reverses fibrinogen’s inhibitory effects on neurite outgrowth (Schachtrup et al., 2007). These findings shed light on fibrinogen’s impact on neuronal development. Moreover, fibrinogen directly interacts with neurons, leading to the upregulation of inflammatory cytokines like CCL2 and IL-6, as well as the activation of the NF-κB transcription factor, promoting a heightened neuroinflammatory response (Sulimai et al., 2021a, 2022). Fibrinogen also stimulates the generation of reactive oxygen species, nitrite, and mitochondrial superoxide in neurons, intensifying oxidative stress and ultimately causing apoptosis and increased neuronal cell death (Sulimai et al., 2021a). Similarly, fibrinogen-induced astrocyte activation has been observed to elevate cell death and apoptosis in co-cultured neurons (Sulimai et al., 2021b). Notably, blocking the function of ICAM-1 or PrP^C^ can mitigate the detrimental effects of fibrinogen on neurons.

In multiple sclerosis (MS) plaques, fibrinogen has been observed to be deposited around blood vessels and bind to CD11b/CD18 receptors on microglia. This binding directly activates microglia, initiating a signaling cascade involving Akt, RhoA, and phosphoinositide 3-kinase (PI3K), leading to changes in cell size, morphology, and enhanced phagocytosis. It promotes microglia differentiation into phagocytic cells. Conversely, depletion of fibrinogen inhibits microglia activation *in vivo* and attenuates inflammatory demyelination (Adams et al., 2007a). Animal studies on MS models have revealed that fibrinogen can contribute to neuroinflammation and axonal injury by inducing the clustering of microglia around blood vessels and the excessive release of ROS and NO intermediates through CD11b/CD18 binding (Davalos et al., 2012). Injection of fibrinogen into the corpus callosum has also been found to stimulate T-cell recruitment and inflammatory demyelination. Moreover, fibrinogen can activate antigen-presenting cells (APCs) via CD11b/CD18 receptors, leading to transcriptional M1-like activation and increased levels of CXCL10 and CCL2. Consequently, this recruitment of microglia and macrophages triggers an autoimmune response, Th1 cell differentiation, and exacerbates demyelination (Ryu et al., 2015). Inhibiting the interaction between fibrinogen and CD11b/CD18 has demonstrated the ability to inhibit microglial activation, reduce inflammatory demyelination, and suppress the pro-inflammatory properties of fibrinogen, without interfering with its procoagulant properties.

**Figure 1:**
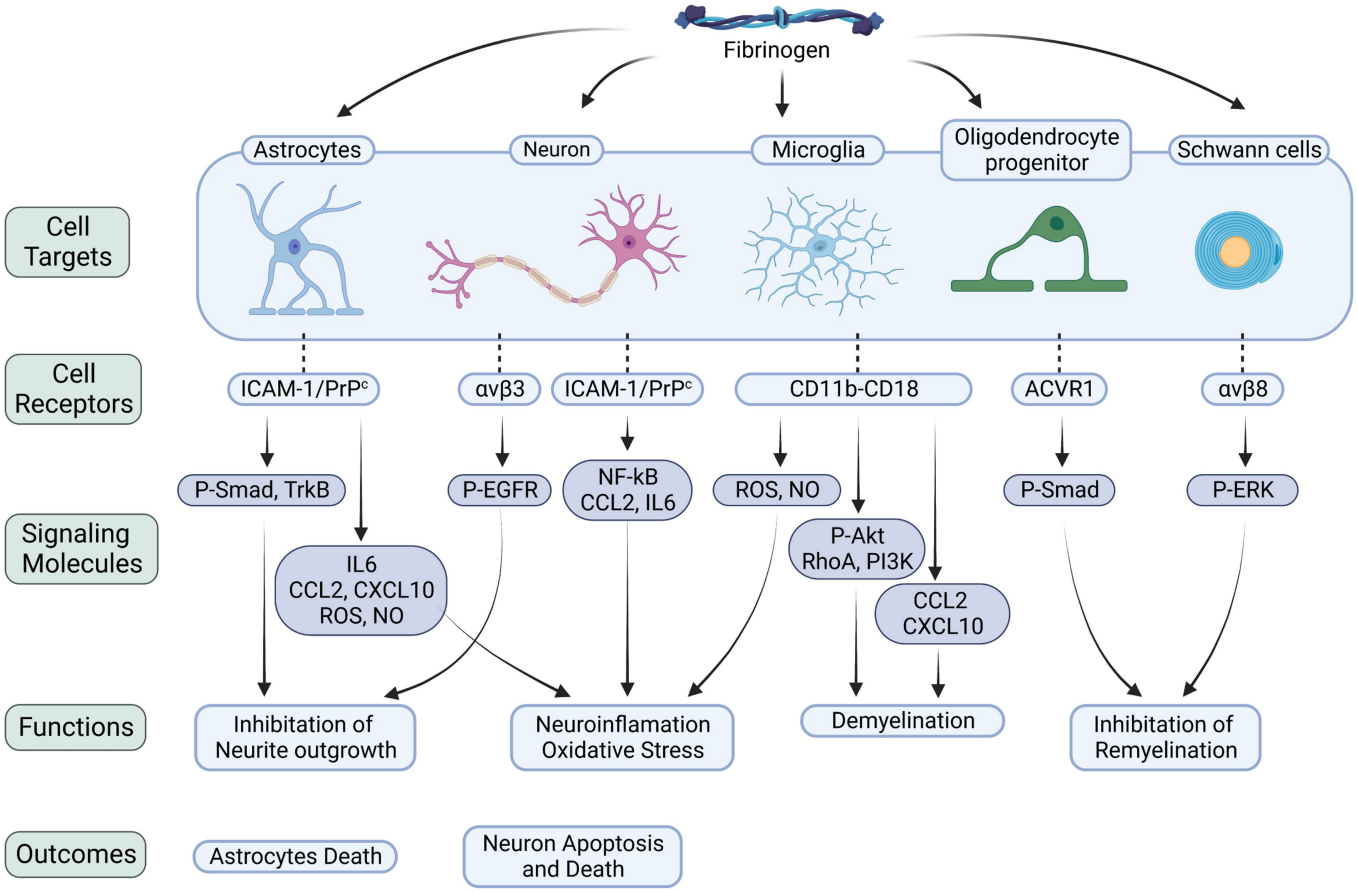
Fibrinogen-related cellular receptors and signaling molecules in the CNS. Fibrinogen plays a critical role in neuroinflammation, neurodegeneration, and myelin damage by binding to receptors on various cells within the nervous system. The interactions with astrocytes, neurons, microglia, OLs, and Schwann cells trigger downstream signaling pathways that worsen neuroinflammatory processes, oxidative stress, and cellular demise. Specifically, when fibrinogen binds to astrocytes, it activates the Smad pathway. This activation leads to the release of inflammatory factors and suppression of neurite growth. Similarly, binding to neurons activates the EGFR, inhibiting neurite growth, and triggers the NF-κB pathway, upregulating the levels of CCL2 and IL6, which result in neuronal death. During its conversion to fibrin, fibrinogen exposes the γ^377–395^ epitope. This epitope binds to the CD11b/CD18 integrin receptor on microglia, promoting the release of ROS and NO, triggering the signaling cascade of the Akt, RhoA, and PI3K pathways, and increasing the levels of CCL2 and CXCL10. Activation of these signaling molecules exacerbates neuroinflammation and demyelination. Furthermore, fibrinogen’s interaction with OLs increases phosphorylation of the BMP signal transducers Smad1/5, impeding myelin regeneration. Its interaction with Schwann cells, on the other hand, stimulates the ERK pathway, also impeding myelin regeneration. P-Smad, phosphorylated mad homolog; P-EGFR, phosphorylated epidermal growth factor receptor; P-Akt, phosphorylated serine/threonine-protein kinase; The integrin receptors: αvβ3, αvβ8; ACVR1, activin A receptor type 1; NF-κB, nuclear factor-κB.

Fibrinogen’s interaction with the ACVR1 receptor on oligodendrocyte progenitor cells (OPCs) has been shown to activate the BMP signaling pathway, inhibiting OPC differentiation into mature myelin oligodendrocytes (OLs) and impairing remyelination (Petersen et al., 2017; Hou et al., 2023). Moreover, the presence of fibrin fibrils or intracellular accumulation of fibrinogen within OLs can induce autophagic stress, leading to OL and myelin loss, as well as axonal degeneration (Montagne et al., 2018). Investigations have also revealed that fibrin plays a role in modulating Schwann cell behavior following nerve injury. Binding of fibrin to Schwann cells through αMβ2 integrin triggers the activation of the ERK1/2 signaling pathway, inhibiting myelin protein synthesis and fibronectin production and maintaining Schwann cells in a non-myelinating state. Interestingly, fibrin removal by the fibrinolysis system promotes Schwann cell transition to a myelinating state, facilitating accelerated axonal remyelination (Akassoglou et al., 2000, 2002). These findings underscore the crucial role of fibrin deposition in exacerbating axonal injury and suggest that the regulation of fibrin clearance and deposition may serve as a key regulatory mechanism for Schwann cell differentiation following nerve injury (Figure 1).

## 4. Effects and mechanisms of fibrinogen in neurodegenerative diseases

Accumulating evidence supports the detrimental role of fibrinogen in exacerbating neuropathological features observed in various neurodegenerative disorders. Conversely, reducing fibrinogen levels has shown promise in alleviating cognitive impairment associated with these diseases. Neurodegenerative disorders share common characteristics such as oxidative stress, neuroinflammation, and damage to both the CNS and peripheral nervous system (Kim et al., 2015; Teleanu et al., 2022). In patients with Alzheimer’s disease (AD) (Fiala et al., 2002), MS (Yates et al., 2017), Parkinson’s disease (PD) (Gray and Woulfe, 2015), vascular dementia (VaD) (Sun et al., 2020), Huntington’s disease (HD) (Drouin-Ouellet et al., 2015), and amyotrophic lateral sclerosis (ALS) (Winkler et al., 2013), fibrinogen and its derivative, fibrin, have been detected in brain deposits. Excessive fibrinogen deposition induces proinflammatory effects, including vasoconstriction, increased vascular permeability, and activation of microglia, astrocytes, and neurons (Jenkins et al., 2018; Li et al., 2019). Extensive research has been conducted to elucidate the role of fibrinogen in different neurodegenerative disorders. In this comprehensive review, we delve into the effects and underlying mechanisms of fibrinogen in various neurodegenerative diseases.

### 4.1. Fibrinogen and AD

AD is a complex neurodegenerative disorder that significantly impacts the lifespan and quality of life of affected individuals, particularly in old age (Qiu et al., 2009). AD is characterized by a progressive decline in cognitive function and is marked by the accumulation of amyloid-β (Aβ) plaques and Tau protein tangles in the brain, synaptic dysfunction, oxidative stress, and neuroinflammation (Khan et al., 2020). Neuroinflammation has emerged as a crucial contributor to the pathogenesis and progression of AD, garnering considerable attention in recent years. One well-established phenomenon in AD is the accumulation of fibrin(ogen) within the brain, predominantly around cerebral blood vessels, often associated with cerebral amyloid angiopathy and perivascular brain tissue (Cortes-Canteli et al., 2010; Hultman et al., 2013). The deposition of fibrin(ogen) is closely linked to key pathological features of AD, including the presence of amyloid plaques, activated microglia, pericyte loss, and dystrophic neurites (Fiala et al., 2002; Ryu and McLarnon, 2009; Sengillo et al., 2013; Cortes-Canteli et al., 2015; Miners et al., 2018). Moreover, elevated levels of fibrinogen have been observed in the plasma and cerebrospinal fluid of AD patients and have been correlated with brain atrophy (Petersen et al., 2018; Strickland, 2018). Animal models of AD have also demonstrated the deposition of fibrin in the central nervous system, serving as a hallmark of progressive neurodegeneration in models with pericyte defects or APOE gene transgenic expression (Montagne et al., 2017).

Fibrinogen extravasation into the brain parenchyma can lead to fibrin deposition, which is associated with neurodegeneration and can have a direct impact on cognitive function. Studies have shown that fibrinogen is co-localized with areas of synaptic dysfunction in AD patients, and reducing fibrinogen levels can improve synaptic function and reduce Aβ deposition and neuronal death in mouse models of AD, such as TgCRND8 mice (Cortes-Canteli et al., 2015). These findings suggest that targeting fibrinogen may be a potential therapeutic approach for AD. Furthermore, fibrinogen has been shown to activate microglia and induce dendritic loss and spine elimination, leading to neuroinflammation, synaptic defects, and cognitive decline in the 5 × FAD mice model via CD11b/CD18 receptors (Merlini et al., 2019). Interestingly, AD mice with impaired fibrin degradation due to a lack of tissue plasminogen activator (tPA) or only one functional plasminogen gene showed aggravated Aβ plaque deposition, vascular damage, and cognitive impairment (Paul et al., 2007; Oh et al., 2014). In contrast, AD mice with only one functional fibrinogen gene had decreased blood–brain barrier (BBB) damage (Paul et al., 2007).

Research has demonstrated that the interaction between fibrinogen and Aβ can have detrimental effects on the brain. Specifically, the central region of Aβ can bind to certain regions of the fibrinogen protein (Zamolodchikov et al., 2016a), leading to the formation of tighter fibrin networks that are more resistant to degradation. This, in turn, can promote the formation of Aβ fibers and increase the spatial extent and duration of fibrin clots in the brain (Ahn et al., 2010; Cortes-Canteli et al., 2010; Zamolodchikov and Strickland, 2012). These changes can contribute to fibrin-induced neurodegeneration and microglial activation, ultimately exacerbating the pathology of AD (Zamolodchikov and Strickland, 2012; McLarnon, 2021). It has been found that Aβ can interacts with coagulation factor XII to activate coagulation factor XI, resulting in the generation of thrombin and the formation of fibrin clots (Zamolodchikov et al., 2016b). Aβ can also enhance high molecular weight kininogen (HK) cleavage through an FXII-dependent pathway (Bergamaschini et al., 2001). In a study using ASO-mediated mRNA knockdown to deplete FXII, it was shown that inhibiting HK cleavage improved neuroinflammation, fibrin(ogen) deposition, neuronal degeneration, and cognitive function in AD mice (Chen et al., 2017). These findings suggest that the accumulation of fibrin(ogen) after vascular damage in AD is a crucial pathogenic component that exacerbates the inflammatory process, contributing to the onset and progression of the disease.

### 4.2. Fibrinogen and MS

MS is a chronic autoimmune, inflammatory, and neurodegenerative disorder that manifests as motor and sensory dysfunction, as well as visual impairment. The disease is characterized by widespread damage to the BBB, inflammation surrounding blood vessels, nerve degeneration, axonal injury, and demyelination, which occur in multiple areas over time (Murua et al., 2022). Notably, disruptions in the BBB and the deposition of fibrin are commonly observed in the white matter of individuals with MS and in experimental autoimmune encephalomyelitis (EAE) mouse models (Gveric et al., 2001; Vos et al., 2005; Marik et al., 2007; Davalos et al., 2012, 2014; Maggi et al., 2014). Studies have demonstrated the presence of fibrin(ogen) deposition in MS lesions, encompassing both the relapsing–remitting and progressive forms of the disease. In the relapsing–remitting form, fibrin is primarily localized in regions of active demyelination and often associates with microglia/macrophages and perivenous demyelination. Conversely, in progressive forms, fibrin exhibits a diffuse distribution and overlaps with astrocyte and axonal processes in chronically active and inactive lesions (Vos et al., 2005; Marik et al., 2007). Moreover, fibrin deposition has been linked to early lesions, inflammatory responses, and areas of axonal damage in MS. Experimental injections of fibrinogen into the corpus callosum or the ventral column of the spinal cord induce inflammatory demyelination, while injections into the cortex lead to dendrite and spine loss (Ryu et al., 2015; Merlini et al., 2019). These findings suggest that the pathological effects of fibrinogen in the brain may be region-specific. Interestingly, fibrin deposition, microglial activation, and mild axonal damage have been observed in MS model mice prior to T cell infiltration, indicating that fibrin(ogen) may serve as a predictive and critical signaling molecule in the development of MS pathology (Marik et al., 2007; Ahmad and Frederiksen, 2020). These insights contribute to our understanding of the role of fibrin(ogen) in the progression of MS and its potential as a target for therapeutic interventions.

A case–control study conducted in Campania, Italy, has provided insights into the potential genetic factors associated with MS. The study revealed a possible association between the FGB 455 G/A variants and MS status, particularly in the recessive model (Abbadessa et al., 2022). These findings highlight the importance of genetic investigations in understanding the underlying mechanisms of MS, although further research is needed to validate and expand upon these results. Furthermore, the presence of fibrinogen in extracellular vesicles (EVs) found in the plasma of individuals with MS has been identified. Notably, the presence of fibrinogen within EVs has been implicated in the occurrence of spontaneous relapsing disease activity in a mouse model of MS (Willis et al., 2019). The activation of the BMP signaling pathway via fibrinogen binding to the ACVR1 receptor in OPCs inhibits oligodendrocyte differentiation and axonal myelination (Petersen et al., 2017). Disruption of the BBB in animal models of MS can lead to increased coagulation activity and the deposition of fibrin in the brain. Fibrinogen binding to CD11b/CD18 integrin receptors not only activates microglia to function as phagocytes (Adams et al., 2007a), but also promotes the recruitment of T cells and the release of pro-inflammatory signals, ultimately resulting in demyelination and axonal injury (Akassoglou et al., 2004; Davalos et al., 2012; Ryu et al., 2015). Antagonizing the interaction between fibrinogen and CD11b using Fibγ^377–395^ peptide or 5B8 has been shown to reduce microglial activation, axonal damage, ROS generation, and demyelination (Adams et al., 2007a; Ryu et al., 2018). These findings suggest that the entry of fibrinogen into the CNS or the deposition of fibrin can serve as initial triggers for inflammatory demyelination. Fibrinogen-mediated pathology plays a crucial role in the development and progression of MS. Inhibiting fibrinogen could therefore be considered as an upstream therapeutic strategy to prevent myelin damage and promote remyelination.

### 4.3. Fibrinogen and PD

PD is a chronic neurodegenerative disorder characterized by a range of motor symptoms, including bradykinesia, muscular rigidity, and resting tremor, as well as nonmotor symptoms like olfactory dysfunction, cognitive impairment, psychiatric symptoms, and autonomic dysfunction (Bloem et al., 2021). The progressive loss of dopaminergic neurons in the substantia nigra of the midbrain and the presence of abnormal protein aggregates called Lewy bodies, primarily composed of fibrillar α-synuclein, are the underlying causes of PD. In recent years, research has increasingly focused on the role of neuroinflammation in the development and progression of PD (De Virgilio et al., 2016). Studies have suggested a potential link between elevated levels of fibrinogen and an increased risk of PD, particularly in elderly men (Wong et al., 2010). Autopsies of PD patients have also revealed significant perivascular deposition of fibrin(ogen) in the striatum, compared to brains from control subjects (Gray and Woulfe, 2015). Additionally, a controlled clinical study investigating carpal tunnel syndrome observed co-deposition and interaction between fibrinogen and α-synuclein in the transverse carpal ligament (Utrobičić et al., 2014). These findings provide further support for the potential involvement of fibrinogen in the pathogenesis of PD and highlight the significance of neuroinflammation in the progression of the disease.

Dysregulation of inflammatory biomarkers and increased circulating bacterial inflammation in PD indicate the presence of systemic inflammation and dysfunction of the innate immune system. Systemic inflammation is often accompanied by oxidative stress, leading to a general hypercoagulable state. Scanning electron microscopy has revealed an unusual coagulation phenomenon in the blood of Parkinson’s disease patients, characterized by abnormal aggregation of fibrinogen and the formation of fibrin amyloid (Adams et al., 2019). Furthermore, elevated levels of palmitoylation of fibrinogen peptides have been observed, particularly in the cortical region of the brain, in individuals with PD (Cervilla-Martínez et al., 2022). Studies in PDCI have found that intraperitoneal injection of native fibrinogen can cause motor defects in C57BL/6 J mice (Naskar et al., 2022). These findings suggest that fibrinogen may play a crucial role as an inflammatory mediator in the pathogenesis of Parkinson’s disease.

### 4.4. Fibrinogen and VaD

VaD, a type of dementia, is characterized by cognitive impairment resulting from damage to specific regions of the brain due to ischemic or hemorrhagic lesions. The extent and location of vascular lesions determine the cognitive deficits observed in VaD patients (Bir et al., 2021). The contribution of vascular mechanisms to dementia development in the elderly population is increasingly acknowledged. VaD is believed to stem from primary vascular injuries in the CNS, including atherosclerosis-associated thrombosis, fibrinoid necrosis or lipolysis, and chronic cerebral hypoperfusion. These events can trigger oxidative stress, neuroinflammation, and central cholinergic dysfunction in the affected areas, leading to cognitive decline (Meyer et al., 2000; Wang et al., 2016). Elevated plasma fibrinogen levels have been identified as a potential risk factor for all-cause dementia and VaD, particularly in individuals below 80 years of age, with a stronger association observed in populations of European ancestry (Vishnu et al., 2017; Zhou et al., 2020). Targeting fibrinogen reduction may hold promise as a therapeutic strategy to prevent or treat VaD in high-risk patients. Moreover, the FGB-148C/T polymorphism, which affects plasma fibrinogen levels, has been found to impact dementia incidence. Specifically, VaD patients with the TT genotype of FGB-148C/T exhibit higher circulating fibrinogen levels compared to those with the CC and CT genotypes. Thus, regulating fibrinogen gene expression could be a potential therapeutic approach for preventing and treating dementia (Sun et al., 2020). Fibrinogen also exerts a significant influence on microvascular reactivity and permeability. Studies conducted on mice with elevated fibrinogen levels have shown increased cerebral vascular permeability through caveolar protein transcytosis. This condition can promote fibrinogen and PrP^C^ deposition in the brain, leading to vascular remodeling and short-term memory loss (Muradashvili et al., 2016). These findings emphasize the importance of fibrinogen level regulation in preventing age-related vascular cognitive impairments.

### 4.5. Fibrinogen and HD

HD is a debilitating neurological disorder caused by a mutation in the Huntingtin gene. The severity of the disease correlates with the length of the cytoine-adenine-guanine (CAG) repeat in the gene. As HD progresses, patients experience motor, cognitive, mental, and metabolic dysfunction, along with the atrophy of key brain regions such as the striatum, cerebral cortex, and subcortical white matter. Mutant huntingtin exacerbates synaptic dysfunction and mitochondrial toxicity through multiple mechanisms, leading to the loss of spiny neurons in the striatum and cerebral cortex (McColgan and Tabrizi, 2018). Immune activation and brain inflammation have also been identified as significant features of HD (Saba et al., 2022). These mechanisms collectively contribute to the progressive deterioration of neurological function in individuals with HD. Analysis of postmortem brain samples from HD patients has revealed notable increases in blood vessel density and BBB permeability in the putamen, along with a 2.5-fold increase in extravascular fibrinogen compared to controls (Drouin-Ouellet et al., 2015). Studies utilizing the YAC128 mouse model have demonstrated microgliosis and proangiogenic activity, while chronic exposure to lipopolysaccharide (LPS) has been shown to enhance microglia activation and induce neurovascular destruction, resulting in increased BBB permeability and fibrinogen deposition in the striatum (Franciosi et al., 2012). These findings suggest that fibrin(ogen) may play a role in the vascular inflammatory processes underlying HD, although the precise mechanisms involved are still being investigated.

### 4.6. Fibrinogen and ALS

ALS is a devastating neurodegenerative disorder characterized by the selective loss of motor neurons in various regions of the brain and spinal cord. This leads to progressive muscle weakness, atrophy, and ultimately, death within a few years after the onset of symptoms (Goutman et al., 2022). While most cases of ALS are sporadic, a small fraction is inherited, with mutations in the Cu/Zn superoxide dismutase (SOD1) gene accounting for about 20% of familial cases. The pathogenesis of ALS is complex and involves multiple mechanisms, including oxidative stress, neuroinflammation, and impaired protein homeostasis (Rosen, 1993). The blood-spinal cord barrier (BSCB) plays a crucial role in maintaining the homeostasis of the CNS and is implicated in the pathogenesis of ALS, particularly in SOD1 mutant mice and rats. Interestingly, BSCB dysfunction occurs before the onset of motor neuron degeneration and neurovascular inflammation in these animal models, indicating that vascular changes are an important early event in ALS pathology (Zhong et al., 2008; Nicaise et al., 2009; Miyazaki et al., 2011). Consequently, damage occurring prior to the entry of blood-derived toxins into the CNS may be a critical early factor that accelerates motor neuron death in ALS. Further investigations into the integrity of the BSCB in postmortem gray and white matter of medullary and spinal cord tissue from both sporadic and familial ALS patients have revealed significant perivascular fibrin deposition compared to controls (Garbuzova-Davis et al., 2012). Autopsy analyses have also demonstrated the accumulation of fibrin in motor neuron-dense areas of the gray matter, specifically in the anterior horn of the cervical spinal cord, in individuals with both sporadic and familial ALS (Winkler et al., 2013). These findings suggest that fibrin accumulation may play a role in the pathogenesis of ALS and could potentially serve as a diagnostic marker or therapeutic target for this devastating neurodegenerative disease.

## 5. Application of fibrinogen-related treatments in neurodegenerative diseases

Fibrinogen, known for its proinflammatory effects, has been the focus of research in the field of neurodegenerative diseases. Animal studies have provided valuable insights into the potential benefits of reducing fibrinogen levels, which include mitigating neuroinflammation, preserving the integrity of the BBB, reducing cerebral amyloid angiopathy, and minimizing axonal damage (Paul et al., 2007; Ryu and McLarnon, 2009; Cortes-Canteli et al., 2010). Researchers have developed various strategies and genetic tools targeting fibrinogen to combat the pathological progression of neurodegenerative diseases. These approaches encompass a range of interventions, such as depleting fibrinogen, inhibiting its conversion to fibrin, blocking its interaction with specific receptors or proteins, and manipulating specific fibrinogen sequences through genetic modifications in mouse models. These innovative strategies hold great promise in the quest for effective therapies against neurodegenerative diseases. By targeting fibrinogen, researchers aim to intervene in the inflammatory processes and pathological cascades associated with these debilitating conditions.

### 5.1. Deplete fibrinogen

Ancrod is a compound derived from the venom of the Malayan pit viper, *Calloselasma rhodostoma*, which possesses a serine protease activity that can cleave fibrinopeptide A from fibrinogen and produce soluble fibrin degradation products (Dempfle et al., 2000).

Ancrod can effectively reduce fibrinogen levels in the circulation and inhibit neuroinflammation and vascular pathology involved in fibrinogen-mediated damage. In a stereotaxic mouse model, where Aβ was injected into the hippocampus, treatment with Ancrod showed a significant reduction in brain parenchymal fibrinogen levels. This, in turn, led to a reduction in microglial proliferation, neuroinflammation, and protection of hippocampal neurons (Paul et al., 2007; Ryu and McLarnon, 2009). Overall, lowering fibrinogen levels can reduce neuroinflammation and inhibit neuronal and BBB damage. However, mice lacking tPA (tPA^−/−^ mice) exhibited increased fibrin deposition as well as aggravated axonal degeneration and demyelination compared with wild-type (WT) mice after subjected to sciatic nerve crush. Ancrod administration alleviated axonal degeneration in tPA^−/−^ mice. These findings suggest that reducing fibrinogen levels may be an effective strategy for alleviating nerve damage exacerbated by fibrin deposition in tPA^−/−^ mice (Akassoglou et al., 2000). In MS mice model, removal of fibrinogen with Ancrod significantly reduced microglial activation and inflammatory demyelination, with a 7-fold reduction in cerebellar demyelination and a 2-fold reduction in spinal cord demyelination. Moreover, administering Ancrod after the onset of paralytic symptoms improved clinical symptoms, reversed recurrent paralysis, and reduced inflammatory demyelination (Akassoglou et al., 2004; Adams et al., 2007a). In addition, the application of Ancrod in EAE model was able to reduce the number and proliferation of interferon-γ (IFN-γ) CD4 T cells, suggesting that fibrinogen depletion via Ancrod inhibits the effector function of encephalitic T cells and reduces innate immune activation (Ryu et al., 2015). These findings suggest that fibrinogen depletion using Ancrod may be a promising therapeutic strategy for treating EAE and other demyelinating diseases. In the pericyte-deficient mouse model PDGFβ^F7/F7^, Ancrod treatment was able to reduce plasma fibrinogen levels and fibrin (ogen) deposition in white matter, and improve pericyte coverage and BBB integrity (Montagne et al., 2018). In addition, pharmacologic depletion of fibrinogen in mice using Ancrod significantly reduce active TGF-β, Smad2 phosphorylation, glial cell activation, neurocan deposition and neuroinflammation after cortical injury in mice (Schachtrup et al., 2010). The use of Ancrod in the lysolecithin (LPC) focal demyelination model can reduce BMP receptor pathway activation and enhance the ability of myelin regeneration, thus improving CNS injury (Petersen et al., 2017). These findings highlight the potential of Ancrod as a therapeutic agent for various neurological conditions.

Batroxobin, a serine protease extracted from the venom of the South American pit viper *Bothrops atrox* moojeni, has the ability to convert fibrinogen into an insoluble form, resulting in a significant decrease in circulating fibrinogen and the development of afibrinogenemia (Stocker and Barlow, 1976). Studies have shown that Batroxobin treatment in EAE rat models and TMEV-IDD mice models reduces fibrin deposition around spinal cord vessels, plasma fibrinogen levels, and demyelination, resulting in an improvement in clinical signs of disease. Furthermore, Batroxobin treatment has been shown to prevent the “opening of the BBB” and inhibit the migration of inflammatory cells from the CNS vasculature to the parenchyma (Inoue et al., 1996, 1997). These results suggest that Batroxobin can effectively inhibit the pathological progression of EAE and TMEV-IDD by preventing fibrin deposition.

### 5.2. Inhibit the conversion of fibrinogen to fibrin

Hirudin is an acidic polypeptide that is secreted by the salivary glands of the medicinal leech *Hirudo medicinalis*. This potent natural substance is a specific and powerful inhibitor of thrombin, the key enzyme involved in blood clotting. Hirudin has been shown to effectively block the conversion of fibrinogen to insoluble fibrin, thereby exerting strong anticoagulant and anti-thrombotic effects (Wakui et al., 2019). In EAE mice, hirudin treatment significantly reduced the formation of perivascular clusters and fibrin deposition, as well as demyelination, as observed by *in vivo* imaging of the same spinal cord segments and correlated histopathological analysis (Davalos et al., 2012). Moreover, hirudin effectively reduced immune cell proliferation and the secretion of cytokines such as IL-6, IL-17, and TNF, ultimately mitigating the severity of EAE. However, the increased risk of bleeding and the production of anti-hirudin antibodies in the serum of EAE mice after hirudin treatment reduce its effectiveness and may lead to side effects (Han et al., 2008). Therefore, while hirudin shows promise as a potential treatment for MS patients, it may not be the optimal choice.

Dabigatran, a newer oral anticoagulant [109], has emerged as a promising therapy in clinical practice for its ability to inhibit the formation of thrombin and fibrin, which promote inflammatory response and are thought to potentially relieve the clinical symptoms of AD patients (Grossmann, 2020, 2021; Iannucci et al., 2020). Studies have demonstrated that the use of dabigatran can effectively reduce the expression of vascular inflammatory proteins, mitigate the generation of ROS, and reduce glial activation in a mouse model of AD (Tripathy et al., 2013; Marangoni et al., 2016). Furthermore, long-term treatment of TgCRND8 mice with Dabigatran significantly reduced amyloid load, fibrin deposition in the brain parenchyma, and prevented occlusive thrombosis, thereby alleviating neuroinflammation, BBB damage, and cognitive impairment in AD mice models (Cortes-Canteli et al., 2019). These findings suggest that Dabigatran may have potential as a therapeutic option for the treatment of AD, and further clinical trials are warranted to validate its efficacy in humans.

### 5.3. Inhibit the interaction of fibrin(ogen) with specific receptors or proteins

The conversion of fibrinogen into fibrin results in the unmasking of an epitope harbored on the γ chain of fibrinogen, specifically the γ^377–395^ amino acid sequence. This exposed epitope has the capability to interact with the CD11b-I domain located on the CD11b/CD18 receptor (Lishko et al., 2002; Ugarova et al., 2003). Studies have found that mice carrying the Fibγ^390–396A^ allele genetically inhibit the fibrinogen-CD11b/CD18 interaction, microglial activation, and perivascular microglial cluster formation, which play a role in inhibiting axonal injury and recurrent paralysis (Davalos et al., 2012). Moreover, mice injected with plasma derived from Fibγ^390–396A^ mice showed 70% less demyelination compared to plasma derived from WT, indicating that inhibiting the interaction of fibrin with CD11b/CD18 can reduce demyelination (Ryu et al., 2015).

Administration of the Fibγ^377–395^ peptide can also interrupt the interaction between fibrinogen and the CD11b/CD18 receptor. *In vivo* studies have demonstrated that vaccination with the Fibγ^377–395^ peptide before the induction of EAE or intranasal administration of the Fibγ^377–395^ peptide after the onset of EAE can significantly reduce microglia activation, inflammatory demyelination, and improve the symptoms of EAE (Adams et al., 2007a). These findings suggest that inhibiting the interaction of fibrin with the CD11b/CD18 receptor may be a potential therapeutic target for demyelinating diseases such as EAE.

In AD models, intranasal administration of the Fibγ^377–395^ peptide has also been shown to improve cognitive performance and reduce Aβ deposition in the cerebral cortex (Aso et al., 2015). The use of stereotaxic injection to administer fibrinogen into the corpus callosum of CD11b-deficient (Itgam^−/−^) mice and WT mice resulted in a marked disparity in the level of microglial activation, T-cell infiltration, demyelination, and the expression of CXCL-10, CCl2, T-bet, IFN-γ, and IL-12p40. Specifically, Itgam^−/−^ mice exhibited a significant reduction in these pathological manifestations compared to their WT counterparts (Ryu et al., 2015). This intriguing observation supports the notion that blocking the fibrinogen-CD11b interaction has the potential to ameliorate the aberrant activation of the adaptive immune response induced by fibrinogen. In addition, the use of anti-MAC-1 (CD11b/CD18) antibody has been found to significantly reduce microglial activation in a mouse model with dual stimulation of Aβ^1–42^ and fibrinogen (Ryu and McLarnon, 2009).

In animal models of MS and AD, a monoclonal antibody called 5B8, which targets the recessive epitope γ^377–395^ of fibrinogen, has been found to selectively bind to fibrin in the brain parenchyma, inhibiting the binding of fibrin with CD11b/CD18 receptors. Importantly, 5B8 did not interfere with fibrin polymerization in human plasma or affect clotting time *in vivo* or partial thromboplastin time (APTT). 5B8 effectively inhibited fibrin-induced NADPH oxidase activation, ROS release, microglial and macrophage activation, and neurodegeneration, leading to reduced innate immune activation and neurodegeneration without comprehensively suppressing innate immunity or interfering with coagulation in various neurological disorders (Ryu et al., 2018). These findings suggest that inhibitory peptides or antibodies targeting the fibrinogen-CD11b/CD18 interaction may selectively inhibit the inflammatory response of fibrinogen in the CNS while preserving its benefits in coagulation, providing a promising treatment strategy for neurodegenerative diseases.

The study suggests that the interaction between fibrinogen and Aβ in AD can exacerbate neuropathology by promoting Aβ plaque formation and abnormal fibrin clots. To address this, researchers screened for a compound (RU-505) that selectively inhibits the fibrinogen-Aβ interaction using high-throughput screening. RU-505 could restore altered thrombosis and fibrinolysis induced by Aβ *in vitro*. *In vivo* studies on Tg6799 mice showed that RU-505 could prevent altered thrombosis and fibrinolysis without affecting normal brain function in WT littermates. Furthermore, long-term treatment with RU-505 could reduce vascular amyloid deposition, fibrinogen infiltration, and microglia proliferation in the cortex of Tg6799 mice, leading to a reduction in neuroinflammation, improved cognitive function, and decreased BBB damage in AD animals (Ahn et al., 2014). These findings suggest that RU-505 could be a potential therapeutic strategy for treating AD by targeting the fibrinogen-Aβ interaction.

As previously mentioned, fibrinogen also shows a strong correlation with astrocytic ICAM-1 or PrP^C^, which can further promote the activation of NF-κB, the overexpression of pro-inflammatory cytokines, and overproduction of ROS and NO in a dose-dependent manner, resulting in neuronal apoptosis and death. However, these effects were ameliorated by treatment with ICAM-1 antibody or PrP^C^ functionally blocking peptide in astrocytes and neurons, indicating that blocking the function of the fibrinogen receptors ICAM-1 and PrP^C^ could be a potential treatment for neurodegenerative diseases with chronic inflammation and substantial fibrinogen deposition in the brain (Sulimai et al., 2021a,b, 2022). In addition, the application of TGF-β receptor inhibitors was able to block the interaction between fibrinogen and TGF-β, eliminating the effect of fibrinogen-induced glial scar formation *in vivo* and *in vitro* (Schachtrup et al., 2010). These findings suggest that targeting the interaction between fibrinogen and its receptors may provide a novel therapeutic approach for neurodegenerative diseases associated with neuroinflammation and neurodegeneration.

### 5.4. Knock out or knock in specific fibrinogen sequences in mice genetic systems

Animal studies have demonstrated that genetic depletion of fibrinogen (fib^−/−^) can reduce the neuroinflammatory response and improve the inhibitory environment after traumatic injury in CNS. Specifically, C57BL/6 J (fib^−/−^) mice showed reduced activation of glial cells and neurocan deposition after cortical injury (Schachtrup et al., 2010). Additionally, in a mouse model of AD, genetic reduction of fibrinogen was found to alleviate neurovascular pathology, BBB damage, and cognitive impairment. Specifically, TgCRND8(fib^+/−^) mice with only one functional fibrinogen gene showed improvements in these pathological features compared to control mice (Paul et al., 2007; Cortes-Canteli et al., 2010). Genetic depletion of fibrinogen showed positive effects in promoting remyelination, reducing inflammation, and delaying the progression of demyelination in mouse models of MS. Specifically, fib^−/−^ mice exhibited faster transition of Schwann cells to a myelin state, leading to faster remyelination of axons and reduced inflammatory demyelination (Akassoglou et al., 2002). Similarly, TgK21p55(fib^−/−^) mice demonstrated reduced expression of major histocompatibility complex class I antigens, neuroinflammation and demyelination, and longer lifespan compared with TgK21(fib^+/+^) mice (Akassoglou et al., 2004). Besides, fibrinogen has been shown to impact the onset of TNF-induced inflammatory demyelination, while axonal injury is associated with increased fibrin (ogen) deposition. Axonal demyelination was aggravated in a mice model of sciatic nerve injury that lack of tPA or plasminogen. The addition of a plasminogen gene defect (plg^−/−^) to a fibrinogen gene defect (fib^−/−^) was able to rescue the worsening of axonal degeneration observed in the plasminogen deficient (plg^−/−^) mice (Akassoglou et al., 2000). These results suggest that axonal injury is associated with increased fibrin (ogen) deposition and tPA/plasmin mediated fibrin (ogen) degradation protects axon invariance and demyelination. As previously highlighted, the Fibγ^390–396A^ allele in mice has the remarkable ability to specifically disrupt the interaction between fibrinogen and the CD11b/CD18 receptor, thus preventing CD11b/CD18 receptor-mediated inflammation while concurrently maintaining normal levels of fibrinogen and coagulation function. Notably, this genetic modification was shown to mitigate microglial activation, perivascular microglia accumulation, nerve degeneration, axonal injury, demyelination, and clinical symptoms of EAE (Flick et al., 2004; Adams et al., 2007a; Davalos et al., 2012). This compelling finding underscores the therapeutic potential of targeting the fibrinogen-CD11b/CD18 pathway in combating age-related neurological disorders (Table 1).

**Table 1:** Fibrinogen-related treatments of neurodegenerative diseases.

Deplete fibrinogen			
Fibrinogen related treatments	Model	Effects	Reference
Ancrod	C57BL/6 J mice	Reduce astrocyte activation, neurocan deposition and neuroinflammation	Schachtrup et al. (2010)
	C57BL/6 mice	Accelerate remyelination	Petersen et al. (2017)
	C57BL/6 J(tPA^−/−^) mice	Reduce axonal damage and muscle atrophy	Akassoglou et al. (2000)
	TgCRND8 mice	Reduce microglia activation	Paul et al. (2007)
	Sprague–Dawley rats	Reduce microglia activation and BBB damage	Ryu and McLarnon (2009)
	SJL/J or C57BL/6 mice	Reduce microglia activation and demyelination	Adams et al. (2007a)
	SJL/J mice	Reduce innate immune activation	Ryu et al. (2015)
	Pdgfrβ^F7/F7^ mice	Reduce fibrin deposition and BBB damage	Montagne et al. (2018)
	Tg6074 mice	Reduce demyelination and immune response	Akassoglou et al. (2004)
Batroxobin	Lewis rats	Reduce fibrin deposition	Inoue et al. (1996)
	SJL/J or C57BL/6 mice		Inoue et al. (1997)
*Inhibit the conversion of fibrinogen to fibrin*			
Hirudin	Cx3cr1^GFP/+^Thy1-CFP mice	Reduce fibrin deposition and demyelination	Davalos et al. (2012)
	SJL/J mice	Reduce immune cell proliferation and cytokine secretion	Han et al. (2008)
Dabigatran	3xTgAD—LaFerla mice	Reduce ROS levels and cerebrovascular expression of inflammatory proteins	Tripathy et al. (2013)
	5 × FAD mice	Reduce microglia activation	Marangoni et al. (2016)
	TgCRND8 mice	Reduce amyloid and fibrin deposition Reduce neuroinflammation	Cortes-Canteli et al. (2019)
*Inhibit the interaction of fibrin(ogen) with specific receptors or proteins*			
Fibγ^377–395^ peptide	AβPP/PS1 mice	Reduce Aβ deposition and cognitive impairment	Aso et al. (2015)
	SJL / J or C57BL/6 mice	Reduce microglia activation and dyskinesia	Adams et al. (2007a)
5B8	Cx3cr1^GFP/+^Ccr2^RFP/+^ mice 5 × FAD mice	Reduce NADPH oxidase activation, ROS release, microglia activation and innate immune activation	Ryu et al. (2018)
Fib^γ390–396A^ allele	Cx3cr1^GFP/+^Fib^γ390–396Α^ mice	Reduce microglia activation, perivascular microglia cluster formation and axonal injury	Davalos et al. (2012)
CD11b-deficient (Itgam^−/−^)	C57BL/6 J(Itgam^−/−^) mice	Reduce microglia activation, demyelination and innate immune activation	Ryu et al. (2015)
Anti-MAC-1 antibody	Sprague–Dawley rats	Reduce microglial activation and loss of neurons	Ryu and McLarnon (2009)
RU-505	Tg6799 mice TgCRND8 mice	Reduce amyloid deposition and microglia proliferation	Ahn et al. (2014)
ICAM-1 antibody or PrP^C^ blocking peptide	Cell(astrocytes and cortical neurons)	Reduce death of astrocyte and neuron	Sulimai et al. (2021a,b, 2022)
TGF-β receptor inhibitor	C57BL/6 J mice	Reduce astrocyte proliferation and glial scar formation	Schachtrup et al. (2010)

Knock out or knock in specific fibrinogen sequences in mice genetic systems		
Model	Effects	Reference
C57BL/6 J(fib^−/−^)mice	Accelerate remyelination Reduce inflammation and inflammatory demyelination	Akassoglou et al. (2002)
C57BL/6 J(fib^−/−^)mice	Reduce astrocyte activation, neurocan deposition and neuroinflammation	Schachtrup et al. (2010)
C57BL/6 J(plg^−/−^ and fib^−/−^)mice	Reduce the worsening of axonal degeneration	Akassoglou et al. (2000)
TgCRND8(fib^+/−^)mice	Reduce neurovascular pathology and BBB damage	Paul et al. (2007)
TgCRND8(fib^+/−^)mice	Reduce CAA pathology and cognitive impairment	Cortes-Canteli et al. (2010)
Fib^γ390–396A^ mice	Reduce microglial activation, perivascular microglia aggregation Reduce nerve degeneration, axonal injury and demyelination	Adams et al. (2007a), Davalos et al. (2012), Flick et al. (2004)
TgK21p55(fib^+/−^)mice	Reduces neuroinflammation and demyelination	Akassoglou et al. (2004)

## 6. Conclusion

As society ages, the incidence of neurodegenerative diseases has increased significantly, severely impacting the elderly’s quality of life. Current treatment strategies mainly aim to alleviate symptoms and slow pathological progression. Symptomatic treatment focuses on reducing neurotransmitter loss and neuroinflammation, while disease-modifying treatments target pathological protein deposition and neuronal death (Trippier et al., 2013; Valera and Masliah, 2013). Despite recent advancements, the efficacy of existing treatment options for neurodegenerative diseases is still limited by their inability to address issues (Huang et al., 2020). As such, there is a burgeoning interest in investigating the contribution of inflammatory factors, such as fibrinogen, to the pathogenesis of neurodegenerative diseases. This avenue of research holds great promise as it could pave the way for the identification of novel therapeutic targets and the development of more effective treatment options.

Evidence from animal and human studies investigating a diverse range of neurodegenerative diseases, including AD, MS, PD and VaD, suggests that fibrinogen is frequently deposited in the brain of affected individuals. Moreover, research has indicated that fibrinogen plays a crucial role in pathogenesis by binding to receptors on nervous system cells, thereby exacerbating oxidative stress, inflammatory activity, neuronal loss, and demyelination. These findings highlight the critical role of fibrinogen in the development and progression of neurodegenerative diseases. Although recent studies have advanced our understanding of the pathological mechanisms underlying neurodegenerative diseases, further research is needed to fully comprehend the role of fibrinogen in the development and exacerbation of clinical symptoms in patients.

Encouragingly, research has demonstrated that drugs capable of reducing plasma fibrinogen levels or inhibiting its interaction with inflammation-related receptors hold significant potential as effective therapeutic interventions to mitigate the pathological progression of neurodegenerative diseases. A retrospective study conducted on Swedish patients with atrial fibrillation demonstrated a notably lower risk of dementia with the use of warfarin or novel oral anticoagulants (Friberg and Rosenqvist, 2018). However, it is important to acknowledge that simply lowering plasma fibrinogen levels may interfere with the crucial role of fibrinogen in coagulation function, consequently increasing the risk of hemorrhagic disorders. An epidemiological survey investigating the use of oral anticoagulants in patients with AD and non-AD patients revealed a gradual decrease in the proportion of patients utilizing oral anticoagulants 2 years after an AD diagnosis, potentially influenced by factors such as the heightened risk of falls in patients (Ali Babar et al., 2022). Particularly in elderly patients, AD brains are prone to hemorrhage due to the presence of cerebral amyloid angiopathy (CAA) (Thal et al., 2008; Smith and Greenberg, 2009; Attems et al., 2011). Therefore, it is crucial to explore and develop fibrinogen-targeting drugs that can effectively impede the pathological progression of neurodegenerative diseases without impacting coagulation function. As of now, there have been limited clinical trials investigating the efficacy of fibrinogen-targeted agents in neurodegenerative diseases. Consequently, well-designed clinical trials are urgently needed to investigate the potential of these drugs in alleviating the clinical symptoms experienced by patients with neurodegenerative diseases.

## Author contributions

TW conceived and wrote the manuscript. ZZ reviewed, edited, and revised the manuscript. All authors contributed to the article and approved the submitted version.

## Funding

This review was supported by the National Research Foundation of China (Grant No. 82071183) to ZZ.

## Conflict of interest

The authors declare that the research was conducted in the absence of any commercial or financial relationships that could be construed as a potential conflict of interest.

## Publisher’s note

All claims expressed in this article are solely those of the authors and do not necessarily represent those of their affiliated organizations, or those of the publisher, the editors and the reviewers. Any product that may be evaluated in this article, or claim that may be made by its manufacturer, is not guaranteed or endorsed by the publisher.
